# Brivaracetam use in clinical practice: a Delphi consensus on its role as first add-on therapy in focal epilepsy and beyond

**DOI:** 10.1007/s10072-024-07485-w

**Published:** 2024-04-01

**Authors:** Simona Lattanzi, Valentina Chiesa, Giancarlo Di Gennaro, Edoardo Ferlazzo, Angelo Labate, Angela La Neve, Stefano Meletti, Carlo Di Bonaventura, Consensus Collaborators Group, Daniela Audenino, Giovanni Boero, Vittoria Cianci, Mario Coletti Moja, Eduardo Cumbo, Filippo Dainese, Giuseppe Didato, Elisa Fallica, Alfonso Giordano, Emilio Le Piane, Mariangela Panebianco, Marta Piccioli, Pietro Pignatta, Monica Puligheddu, Patrizia Pulitano, Federica Ranzato, Rosaria Renna, Eleonora Rosati, Stella Vergine

**Affiliations:** 1 Department of Experimental and Clinical Medicine, Neurological Clinic, Marche Polytechnic University, Via Conca 71, 60020 Ancona, Italy; 2 Epilepsy Center, Child Neurology Unit, ASST Santi Paolo Carlo, Milan, Italy; 3 IRCCS Neuromed, Pozzilli, Italy; 4 Department of Medical and Surgical Sciences, Magna Græcia University of Catanzaro, Catanzaro, Italy; 5 Neurophysiopathology and Movement Disorders Clinic, University of Messina, Messina, Italy; 6 DiBraiN, University Hospital of Bari “A. Moro”, Bari, Italy; 7 Department of Biomedical, Metabolic and Neural Science, University of Modena and Reggio Emilia, Modena, Italy; 8 Neurology and neurophysiology unit – AOU Modena, Modena, Italy; 9 Department of Human Neurosciences, Policlinico Umberto I, Sapienza University of Rome, Rome, Italy

**Keywords:** Brivaracetam, Add-on, Epilepsy, Delphi, Consensus

## Abstract

**Background:**

Antiseizure medications remain the cornerstone of treatment for epilepsy, although a proportion of individuals with the condition will continue to experience seizures despite appropriate therapy. Treatment choices for epilepsy are based on variables related to both the individual patient and the available medications. Brivaracetam is a third-generation agent antiseizure medication.

**Methods:**

We carried out a Delphi consensus exercise to define the role of brivaracetam in clinical practice and to provide guidance about its use as first add-on ASM and in selected clinical scenarios. A total of 15 consensus statements were drafted by an expert panel following review of the literature and all were approved in the first round of voting by panelists. The consensus indicated different clinical scenarios for which brivaracetam can be a good candidate for treatment, including first add-on use.

**Results:**

Overall, brivaracetam was considered to have many advantageous characteristics that render it a suitable option for patients with focal epilepsy, including a fast onset of action, favorable pharmacokinetic profile with few drug-drug interactions, broad-spectrum activity, and being well tolerated across a range of doses. Brivaracetam is also associated with sustained clinical response and good tolerability in the long term.

**Conclusions:**

These characteristics also make it suitable as an early add-on for the elderly and for patients with post-stroke epilepsy or status epilepticus as highlighted by the present Delphi consensus.

**Supplementary Information:**

The online version contains supplementary material available at 10.1007/s10072-024-07485-w.

## Introduction

Epilepsy is a complex brain condition resulting from multiple risk factors and complex genetic predisposition, frequently associated with neurobiological, cognitive, psychological, and social consequences [1]. Epilepsy is one of the most common neurological diseases with an incidence that ranges from 40 to 60 per 100,000 inhabitants per year in high-income countries [2]. Focal seizures are the most common type of seizures and are present in over 60% of people with epilepsy [3]. Due to its chronic nature, epilepsy imposes a significant burden on individuals and society. Epilepsy is also associated with increased risk of injury and individuals with epilepsy have a higher risk of premature death compared to the general population [4]. Epilepsy is often associated with comorbidities like depression, anxiety, and cognitive impairment, and people with the condition experience impaired quality of life that is further burdened by adverse events associated with treatment [4].

Antiseizure medications (ASMs) are the mainstay of treatment. Despite the increasing number of drugs that have become available over the last two decades, around one-third of individuals with epilepsy continue to experience seizures despite appropriate treatment [5, 6]. Brivaracetam is a rationally developed compound characterized by high-affinity binding to synaptic vesicle protein 2A (SV2A) and has a chemical structure similar to levetiracetam (LEV) [7]. Brivaracetam is currently approved by the European Medicines Agency as “adjunctive therapy for treatment of partial onset seizures with or without secondary generalization in patients over the age of 2 years with epilepsy” [8] and by the Food and Drug Administration as “monotherapy and adjunctive therapy for the treatment of partial-onset seizures in patients 1 month of age and older” [9].

Treatment choices for epilepsy are based on several variables related to both the individual patient with epilepsy and the available medications [10]. Factors that are more relevant in the selection of an ASM as first add-on treatment include mechanism of action, effectiveness, tolerability, and risk of drug-drug interactions. Given the fact that each patient has specific characteristics that must be taken into consideration, treatment needs to be personalized since there is no specific ASM that is appropriate for all patients [11].

In the absence of specific guidelines for the selection of the best treatment in people with focal seizures, we carried out a Delphi consensus to define the role of brivaracetam in clinical practice and to provide guidance about its use as first add-on ASM and in selected clinical scenarios.

## Materials and methods

### Delphi process

The Delphi method is an iterative process that uses systematic progression of repeated rounds of voting and is an effective process for determining expert group consensus where there is little or no definitive evidence and where opinion and clinical experience is important [12]. The consensus process utilized a three-step Delphi method. Prior to the first step, a literature search was carried out on PubMed to serve as the basis for discussion and to define areas of interest. Publications were limited to the last 10 years; only publications in English or Italian were included. The search string used was (“brivaracetam” [Supplementary Concept] OR brivaracetam OR 2-(2-oxo-4-propylpyrrolidin-1-yl)butanamide OR ucb34714 OR ucb-34714 OR “ucb34714”) AND (“Epilepsy”[Mesh] OR “Seizures”[Mesh] OR Epileps* OR epilept* OR seizure* OR convulsion* OR convulsive) AND (2012:2022[pdat]) AND (english[Filter] OR italian[Filter]). The results of the selection process are shown in Supplementary Fig. 1.

The eight members of the steering committee were chosen based on participation in clinical trials, extensive clinical experience with ASMs, epilepsy experience, and clinical practice at centers of excellence. In the first step, in February 2023, the steering committee defined eight overarching themes on brivaracetam in focal epilepsy and developed 15 consensus statements. In the second step, in May 2023, two independent meetings were carried out, one with four members of the steering committee and nine panelists, and one with the other four members of the steering committee and ten panelists. The panelists were selected based on knowledge and experience with brivaracetam in clinical practice, and were mostly practicing at second-level centers with an outpatient clinic. Only the panelists voted on each statement. Voting was carried out blindly online and panelists were also able to add comments regarding their vote. In the last step, a final meeting of the steering committee was held in June 2023 to collate the results of the two meetings performed in step 2.

### Analysis of voting and determination of agreement

Participants were asked to provide their level of agreement with the statements proposed using a 5-point Likert scale from 1 to 5, where 1 is complete disagreement and 5 is complete agreement. The threshold level for final agreement was set at 75% of responses being 4 and 5 (agree + strongly agree).

## Results

### Overarching themes

The overarching themes developed related to general features such as ease of use, efficacy/effectiveness, tolerability, and safety of brivaracetam (Table 1). The overarching themes were not voted upon since they depict consolidated concepts with solid evidence as described in detail below. Overall, brivaracetam was considered to be a drug that is easy to use, has a favorable tolerability and pharmacokinetic profile [13, 14], requires no titration [8], has a rapid onset of action [15], and is available both as oral and as intravenous formulations [16–18]. The drug demonstrated efficacy in both phase 3 trials and real-life studies [19, 20]. The drug was also considered to have a good safety profile with little potential for drug-drug interactions [19]. In addition, in the experience of the steering committee, people starting therapy with intravenous brivaracetam can remain on the same dose when they are switched to the oral formulation, without any loss of efficacy or dose modification due to the bioequivalence of intravenous and oral formulations. It was also highlighted that brivaracetam appears to be better tolerated than levetiracetam, especially considering psychiatric/behavioral adverse events.

**Table 1:** Overarching themes on brivaracetam in focal epilepsy

Ease of use	1	Brivaracetam is characterized by an easy-to-use profile
	2	Brivaracetam does not require titration and can be started at the target dose
	3	Brivaracetam is available in oral and intravenous bioequivalent and bioavailable formulations and has a rapid entry across the blood–brain barrier
Efficacy/effectiveness	4	The efficacy of brivaracetam as adjunctive therapy for uncontrolled focal-onset seizures in patients with epilepsy has been demonstrated in pivotal phase III trials and real-world studies
	5	Evidence from the literature indicates that brivaracetam has a rapid onset of action (as early as day 1 of treatment)
	6	Evidence from the literature suggests that brivaracetam has sustained efficacy over time
Tolerability and safety	7	Brivaracetam is characterized by a good tolerability and safety profile
	8	Brivaracetam has a favorable pharmacokinetic profile with few clinically relevant interactions with other anti-seizure and non-anti-seizure medications

### Consensus statements

High level of consensus was reached for all 15 statements on the use of brivaracetam in daily practice in the first round of voting (Table 2).

**Table 2:** Consensus statements and results after the first round of voting

Consensus statements		Agreement (%)
S1	Based on efficacy, tolerability and safety data, brivaracetam can be considered as an appropriate treatment option in elderly patients	100
S2	Brivaracetam is a good treatment option in patients suffering with cardiac diseases	100
S3	Brivaracetam is not associated with worsening of behavioral disturbances in patients with intellectual disability and developmental epileptic encephalopathies and may thus be considered in this group of patients	84.2
S4	Brivaracetam may be a therapeutic option in patients with psychiatric comorbidities	78.9
S5	Brivaracetam is a safe and manageable drug in patients with mild to severe renal impairment and end-stage renal disease	84.2
S6	Brivaracetam is a valid therapeutic option in patients with post-stroke epilepsy	94.7
S7	Brivaracetam has broad-spectrum activity and may be useful in cases of uncertainty of whether seizures are focal or generalized	89.5
S8	Brivaracetam is effective in treating generalized seizures	79.0
S9	Intravenous brivaracetam is a valid alternative for the treatment of status epilepticus after failure of first-line therapy (benzodiazepine)	84.2
S10	Intravenous brivaracetam represents a valid alternative for the treatment of acute symptomatic cluster seizures and cluster seizures in patients with known epilepsy	89.4
S11	Brivaracetam can be a valid therapeutic option in patients who did not previously respond to levetiracetam	94.7
S12	Overnight switching from levetiracetam to brivaracetam is a safe procedure	94.7
S13	The occurrence of psychiatric adverse events associated with levetiracetam does not preclude the use of brivaracetam	94.8
S14	Combining brivaracetam with sodium channel blockers is an example of rational polytherapy that enhances efficacy and improves tolerability of treatment	100
S15	Brivaracetam is suitable for use as first add-on treatment considering its pharmacological characteristics and real-world evidence on efficacy and tolerability	100

*Statement 1*: Based on efficacy, tolerability, and safety data, brivaracetam is an appropriate treatment option in elderly patients. This statement is supported by literature data as detailed below. The drug has been shown to have few clinically relevant interactions with other ASMs and non-ASMs [13, 14, 21]. The drug has been shown to be well tolerated in elderly people [20, 22]. In the BRIVAracetam add-on First Italian netwoRk Study (BRIVAFIRST), adverse events with brivaracetam were documented in 24.2% of older compared to 30.8% of younger participants [20]. In BRIVA-LIFE, among the 575 patients enrolled, 57 (9.9%) were over the age of 65 years [23]. Among the older patients, a significantly greater proportion were seizure-free (37.5% vs 15.3%; *p* < 0.001) and responders (60.7% vs 37.4%; *p* < 0.001) at 12 months compared to younger patients with a similar incidence of adverse events (42.1% in the elderly vs. 39.6% in younger patients). In a pooled analysis of brivaracetam for focal seizures in the elderly, efficacy and safety of adjunctive were consistent with that seen in younger patients [22].

Brivaracetam has no adverse effects on cardiac rhythm [24] and no meaningful adverse cognitive effects [25, 26]. There is also no need for dose adjustment in case of renal impairment [8, 27]. In the elderly, brivaracetam can be efficacious at low doses, and slow titration can be advised. The absence of interactions with the oral anticoagulants [21] makes the drug a good option in elderly people who are not taking oral anticoagulants, but who may need to take them in the future given the high risk of atrial fibrillation after the age of 80 years. The IV formulation is also important for elderly patients who are frequently hospitalized, possibly for reasons unrelated to epilepsy.

*Statement 2*: Brivaracetam is a good treatment option in patients suffering from cardiac diseases, since it has no adverse effects on cardiac rhythm/electrophysiology [24]. Brivaracetam has no interactions with oral anticoagulants that can be taken by people suffering from heart diseases.

*Statement 3*: Brivaracetam is not associated with worsening of behavioral disturbances in patients with intellectual disabilities and developmental epileptic encephalopathies. Steinhoff et al. carried out a systematic review of studies reporting on irritability, anger, or aggression with brivaracetam and other ASMs [28]. It was found that the weight mean incidence of irritability, anger, or aggression with brivaracetam was 5.6%, 3.3%, and 2.5%, respectively. In addition to these low incidences overall, it was held that there is real-world evidence showing that switching from levetiracetam to brivaracetam may improve behavioral adverse events. In addition, the drug has been shown to have no significant effects on cognitive function [25, 26, 29–31]. The lack of effects on the cognitive functioning was confirmed in a naturalistic clinical setting wherein 43 patients underwent neuropsychological screening before adjunctive treatment with brivaracetam; in that analysis, objective gains in attention and executive function was found along with self-reported improvements in concentration and comprehension [30].

In a more general perspective, comorbidities can negatively impact the quality of life of people with epilepsy [32] and there is increasing interest in the assessment of the effects that ASMs can have on other aspects of the disease beside seizures [33, 34]. In this regard, a recent analysis of the international EXPERIENCE pooled data assessed the 12-month effectiveness and tolerability of brivaracetam in adults with epilepsy according to specific comorbidities and epilepsy etiologies at baseline [35]. Although some etiologies were poorly represented, brivaracetam prescribed in the real world was effective and well tolerated across a range of people with epilepsies, including those with cognitive/learning disability, psychiatric comorbidity, post-stroke epilepsy, brain tumor–related epilepsy, and traumatic brain injury–related epilepsy [35].

*Statement 4*: Brivaracetam may be a therapeutic option in patients with psychiatric comorbidities.

Psychiatric comorbidities generally increase the risk of psychiatric side effects [36] and the most appropriate ASM for people with psychiatric disorders or at risk of developing behavioral problems needs to be carefully chosen. In the EXPERIENCE study, discontinuation of brivaracetam due to tolerability issues occurred at similar rates in people with and without psychiatric comorbidity and only few participants reported psychiatric adverse events, suggesting that brivaracetam treatment did not exacerbate pre-existing psychiatric disorders [35]. Consistently, a UK observational study reported similar tolerability profile of brivaracetam irrespective of the presence of pre-existing psychiatric or behavioral comorbidities [25].

Brivaracetam has been shown to be safer than levetiracetam. Robust real-world evidence demonstrated that switching from levetiracetam to brivaracetam can improve behavioral side effects [28]. In addition, brivaracetam was associated with lower incidences of irritability and aggression than levetiracetam and perampanel, and the discontinuation rates due to irritability and aggression were lower with brivaracetam compared to levetiracetam, perampanel, and topiramate [28]. It is, however, worth to notice that no direct or indirect evidence exist for other ASM and it is, therefore, not possible to draw definitive conclusions about how brivaracetam compares with other ASMs.

*Statement 5*: Brivaracetam is a safe and manageable drug in patients with mild to severe renal impairment and end-stage renal disease since the dose does not need to be adjusted. Of note, the drug is not recommended in patients with end-stage renal disease undergoing hemodialysis given the lack of data [8, 27]. Severe renal impairment (creatinine clearance < 15–29 mL/min) decreases the renal clearance of brivaracetam by 63%, but since < 10% of the dose is excreted unchanged in the urine, and because brivaracetam is weakly protein bound, renal impairment has only minimal effects on plasma clearance [27].

*Statement 6*: Brivaracetam is a valid therapeutic option in patients with post-stroke epilepsy. The efficacy and tolerability of brivaracetam has been demonstrated in individuals affected by post-stroke epilepsy [37]. A subgroup analysis of the BRIVAFIRST showed that 43% of participants with post-stroke epilepsy had a reduction in baseline seizure frequency by at least 50% and 35% were free from seizures at 12 months from starting treatment [37]. Adverse events were reported by 20% of participants, and were mild in 85% and moderate in 15% of cases [37]. It was also commented by the panelists that brivaracetam is particularly useful in people with cardioembolic stroke taking oral anticoagulants and in those with AV block or tachycardia-bradycardia.

*Statement 7*: Brivaracetam has a broad-spectrum activity and may be useful in cases of uncertainty whether seizures are focal or generalized, even if this use is off-label. In clinical studies, the drug has broad spectrum efficacy on both focal and generalized seizures, with no worsening of other seizure types such as absence or myoclonic seizures [8, 25]. In the EXPERIENCE study, an international, real-world, pooled analysis on 1644 patients with epilepsy (92% with focal-onset seizures), ≥ 50% seizure reduction was achieved by 36.9% of patients at 12 months [38]. Brivaracetam has also been evaluated in a photosensitivity model where it suppressed generalized photoparoxysmal EEG response [39].

*Statement 8*: Brivaracetam is effective in treating generalized seizures [8, 25, 38]. In a study including 134 epileptic individuals with psychiatric comorbidities and intellectual disability who were prescribed brivaracetam as adjunctive therapy, a 50% responder rate was seen in 29% participants with focal epilepsy compared to 47% in those with generalized and combined focal and generalized epilepsies [25]. In this regard, the panelists commented that it may be desirable to prospectively evaluate the use of brivaracetam in clinical practice in generalized, and in particular in myoclonic epilepsies.

Statements 9 and 10 regarded the use of the intravenous formulation of brivaracetam. *Statement 9*: intravenous brivaracetam is a valid alternative for the treatment of status epilepticus after the failure of first-line therapy (benzodiazepine). *Statement 10*: intravenous brivaracetam represents a valid alternative for treatment of both acute symptomatic cluster seizures and cluster seizures in patients with known epilepsy. It should be noted that statements 9 and 10 consider emergency situations for which the drug is not authorized yet.

There is increasing evidence supporting the use of brivaracetam in urgency-emergency situations and in-hospital setting [8, 40–43]. In a study involving 56 subjects (mean age 62 years; 57% male) with status epilepticus, intravenous brivaracetam was effective in 32 (57%) cases [40]. An early response was seen in 22 (39%) cases, and the administration of intravenous brivaracetam within 6 h from the onset of status epilepticus was independently associated with its early resolution. Importantly, there were no severe treatment-related adverse events. A recent real-world study on the effectiveness of intravenous brivaracetam as a second-line treatment in people with status epilepticus was recently published by Martellino et al. [44]. Of the 21 patients, 14 (66.7%) showed a good early response in the subsequent 6 h after administration, while 8 and 11 patients did not present seizures at 12 and 24 h, respectively.

In a randomized, open-label trial of intravenous brivaracetam versus lorazepam for acute treatment of increased seizure activity involving 45 subjects, 11 had a seizure within 12 h of administration of trial medication (lorazepam 5/15 [median time to next seizure, 5.55 h], brivaracetam 100 mg 3/15 [5.97 h], and brivaracetam 200 mg 3/15 [3.60 h]) [41]. Moreover, most patients were seizure-free over 12 h (lorazepam 9/15 [60.0%], brivaracetam 100 mg 12/15 [80.0%], and brivaracetam 200 mg 12/15 [80.0%]). Use of rescue medication within 12 h was numerically higher for lorazepam (6/15 [40.0%]) versus brivaracetam 100 mg (1/15 [6.7%]) and versus brivaracetam 200 mg (2/15 [13.3%]), and treatment-emergent adverse events were seen in 5/16 (31.3%) participants treated with lorazepam, 6/15 (40.0%) with brivaracetam 100 mg, and 3/15 (20.0%) with brivaracetam 200 mg. Thus, brivaracetam at 100 and 200 mg had a similar efficacy as lorazepam in controlling acute seizures.

The intravenous formulation of brivaracetam is associated with rapid onset of action [15] and flexibility of administration with no need for titration [8, 16]. The fast onset of action of brivaracetam, faster than that of levetiracetam, is an important characteristic of the drug in this setting. Importantly, brivaracetam has been shown to have a good safety in emergency conditions. In such situations, brivaracetam should be administered as a bolus over 2 min [8]. For the dosage, 1.82 mg/kg can be considered adequate [45]. It is important to note that brivaracetam has no known clinically relevant interactions with other ASMs and non-ASMs, with the exception of rifampicin [13, 14]: the plasma levels of brivaracetam have been shown to decrease when co-administered with rifampicin. This type of drug-drug interaction, however, could be extended to all drugs that are strong enzyme inducers, like carbamazepine and phenobarbital, and plasma levels of BRV are expected to decrease when it is administered in a patient already taking a strong inducer. This may be even more important during status epilepticus and in emergency situations.

Statements 11 and 12 concerned response to and switching to levetiracetam. *Statement 11*: brivaracetam can be a valid therapeutic option in patients who did not previously respond to levetiracetam. Brivaracetam has been shown to be effective in people with focal epilepsy independently of previous therapy with levetiracetam [17, 20]. In BRIVAFIRST, the rate of seizure freedom was 22% in people naïve to levetiracetam, 7% in people with history of prior levetiracetam use and discontinuation of it due to inadequate efficacy, and 31% in people with prior use of levetiracetam use and discontinuation of it due to adverse events (*p* < 0.001) [20]. Previous or ongoing treatment with levetiracetam does not preclude the use of brivaracetam [46, 47]. *Statement 12*: Overnight switching from levetiracetam to brivaracetam is a safe procedure, even from high doses of levetiracetam. In exploratory study of 29 patients with epilepsy switching from levetiracetam to brivaracetam, no clinical issues were noted with an immediate switch without dose titration [48]. In the BRIVALIFE study, most patients (223/228) taking levetiracetam at baseline switched to brivaracetam [23]. Of these, 81 (36.3%) patients transitioned overnight, while 142 (63.7%) transitioned progressively over a mean of 21.5 days. The mean dose of levetiracetam at initiation of brivaracetam was 1904 mg/day (range 250–4000 mg/day). For patients who transitioned from levetiracetam to brivaracetam, a 1:10 ratio was used for those on a median dose of levetiracetam of ≤ 2000 mg/day; for those on a higher dose, a higher ratio (1:15) was used. More patients who switched overnight experienced seizure worsening compared to those who underwent a progressive transition (8.1% vs 6.9%). On the other hand, those who transitioned progressively reported more adverse events leading to discontinuation (7.7% vs 2.5%).

*Statement 13*: the occurrence of psychiatric adverse events associated with levetiracetam does not preclude the use of brivaracetam. These adverse effects can improve after the switch to brivaracetam. Yates et al. reported that there was a reduction in the maximum intensity of behavioral adverse events in 93% of patients when switching from levetiracetam to brivaracetam [48]. In the study by Zahnert et al. on 93 patients who were switched to brivaracetam from levetiracetam, 45% had psychiatric comorbidities [47]. Levetiracetam-related and behavioral adverse events, including psychosis, were significantly reduced after the switch to brivaracetam.

*Statement 14*: combining brivaracetam with sodium channel blockers is an example of rational polytherapy that enhances efficacy and improves tolerability of treatment. Favorable combinations usually consist of ASMs with different mechanisms of action [49, 50]. Considering the pharmacodynamic properties of brivaracetam, it may be favorably administered with any other ASM except levetiracetam, whose combination is contraindicated. Clinical data exist about the combination of brivaracetam with sodium channel blockers as an example of rational polytherapy. In BRIVAFIRST, there was a significantly higher number of responders to brivaracetam among subjects who were receiving sodium channel blockers compared to subjects not receiving them (*p* = 0.006) [20]. Adverse events were seen in 30.1% of patients, but were less frequent in patients treated with brivaracetam and concomitant SCBs compared to those not receiving a SCB (28.9% vs. 39.8%, respectively). This favorable combination is likely related to the fact that brivaracetam has a mechanism of action different from other ASMs. The combination of brivaracetam with ASMs acting as sodium channels blocker may thus represent a rational therapy in people with focal onset seizures, and deserves to be further explored with additional studies.

*Statement 15*: brivaracetam is suitable for use as the first add-on treatment according to its pharmacological characteristics and real-world evidence on efficacy and tolerability. In BRIVAFIRST, sustained seizure response was achieved by 60.3% of participants in the early (after 1–2 ASMs) add-on group compared to 34% in the late (after ≥ 3 ASMs) add-on group (*p* < 0.001) [31]. Sustained seizure freedom was obtained by 32% of participants in the early add-on group versus 11% in the late add-on group (p < 0.001). Lastly, adverse events were seen in 39% of subjects in the early add-on group compared to 29% of those receiving brivaracetam as late add-on treatment (*p* = 0.017). It is worth noticing that there is no clear-cut evidence supporting the superiority of an ASM when adjusting for influencing factors [51] and treatment needs to be individualized according to a variety of factors related to both the drug and the person with epilepsy.

## Discussion

The present Delphi consensus aimed to provide key guidance for clinicians about the use of brivaracetam in routine practice. The consensus indicated different clinical scenarios for which brivaracetam can be a good candidate for treatment, including first add-on use. Review of the literature revealed that brivaracetam has many favorable characteristics that render it a suitable option for patients with focal epilepsy. It has a fast onset of action with rapid occupation of SV2A in the brain [15]. It also has a favorable pharmacokinetic profile with few drug-drug interactions and can be combined with most direct oral anticoagulants, ASMs, and sodium channel blockers [8, 13, 14, 17, 21, 46]. Brivaracetam has a broad-spectrum activity and was considered to be useful in cases of uncertainty whether seizures are focal or generalized [8, 25, 38] as well as in cases of coexistence of focal and generalized seizures [8, 25, 38]. Consensus was also reached that intravenous brivaracetam is a valid alternative for the treatment of status epilepticus after failure of first-line therapy, even if off-label, that can be used in emergency situations. The drug requires no titration, can be considered easy to use, and is available in different, bioequivalent formulations [8, 16]. Brivaracetam is well tolerated across doses [8, 16, 17] and has no adverse effects on cardiac arrythmia or cognitive function [24–26], and there is no need for dose adjustments in patient with renal impairment [8, 27]. Brivaracetam is also associated with sustained clinical response and good tolerability in the long term [52–55]. These features make it suitable as an early add-on for the elderly and for patients with post-stroke epilepsy or status epilepticus [15, 31, 37, 40, 41, 46].

### Limitations

The limitations of the present consensus exercise are related to inherent drawbacks of the Delphi technique since it uses controlled feedback and ideas are not openly discussed. In addition, the number of voting participants may be relatively small. However, a high level of consensus was reached for all the statements drafted in the first round of voting, which could indicate that there is little controversy regarding their applicability in daily practice according to the experts.

### Clinical relevance

Brivaracetam is suitable for use as first add-on treatment considering both its pharmacological characteristics and real-world evidence on efficacy and tolerability. It should be noted that data is still lacking on use of brivaracetam in some situations such as pregnancy, and as such caution is warranted in young women with reproductive potential [56]. While the choice of which conditions to consider in developing the present statements was based on the information from consolidated literature data (registrational studies, long-term data, and real-world evidence) and with large sample sizes, it should not be overlooked that the drug may also represent a good choice in other situations, such as for example in the treatment of patients with brain tumor–related epilepsy. In summary, the favorable characteristics of brivaracetam were confirmed by the present Delphi consensus with focus on use of the drug in daily practice.

### Supplementary Information

Below is the link to the electronic supplementary material.

Supplementary file1 (DOCX 102 KB)
