# Correction to: Brivaracetam use in clinical practice: a Delphi consensus on its role as first add‑on therapy in focal epilepsy and beyond

**DOI:** 10.1007/s10072-024-07526-4

**Published:** 2024-04-11

**Authors:** Simona Lattanzi, Valentina Chiesa, Giancarlo Di Gennaro, Edoardo Ferlazzo, Angelo Labate, Angela La Neve, Stefano Meletti, Carlo Di Bonaventura, Consensus Collaborators Group

**Affiliations:** 1 Department of Experimental and Clinical Medicine, Neurological Clinic, Marche Polytechnic University, Via Conca 71, 60020 Ancona, Italy; 2 Epilepsy Center, Child Neurology Unit, ASST Santi Paolo Carlo, Milan, Italy; 3 IRCCS Neuromed, Pozzilli, Italy; 4 Department of Medical and Surgical Sciences, Magna Græcia University of Catanzaro, Catanzaro, Italy; 5 Neurophysiopathology and Movement Disorders Clinic, University of Messina, Messina, Italy; 6 DiBraiN, University Hospital of Bari “A. Moro”, Bari, Italy; 7 Department of Biomedical, Metabolic and Neural Science, University of Modena and Reggio Emilia, Modena, Italy; 8 Neurology and neurophysiology unit – AOU Modena, Modena, Italy; 9 Department of Human Neurosciences, Policlinico Umberto I, Sapienza University of Rome, Rome, Italy

**Correction to: Neurological Sciences (2024)**

10.1007/s10072-024-07485-w

The original article contains an error. In the Consensus Collaborators Group, author name has been inverted during the publication. Family name was captured first instead of the given name. The corrected names as follows:

- **Consensus Collaborators Group**: Daniela Audenino, Giovanni Boero, Vittoria Cianci, Mario Coletti Moja, Eduardo Cumbo, Filippo Dainese, Giuseppe Didato, Elisa Fallica, Alfonso Giordano, Emilio Le Piane, Mariangela Panebianco, Marta Piccioli, Pietro Pignatta, Monica Puligheddu, Patrizia Pulitano, Federica Ranzato, Rosaria Renna, Eleonora Rosati, Stella Vergine.

The original article has been corrected.

