# Supplementary material for: Brivaracetam use in clinical practice: a Delphi consensus on its role as first add-on therapy in focal epilepsy and beyond
Source: Neurol Sci. 2024 Apr 1;45(9):4519–27. doi: 10.1007/s10072-024-07485-w (PMC11306259; doi:10.1007/s10072-024-07485-w)

**Brivaracetam use in clinical practice: a Delphi consensus on its role as first add-on therapy in focal epilepsy and beyond**

**Neurological Sciences**

**Authors**: Simona Lattanzi^1^, Valentina Chiesa^2^, Giancarlo Di Gennaro^3^, Edoardo Ferlazzo^4^, Angelo Labate^5^, Angela La Neve^6^, Stefano Meletti^7^, Carlo Di Bonaventura^8^, Consensus Collaborators Group*

**Consensus Collaborators Group:** Audenino Daniela, Boero Giovanni, Cianci Vittoria, Coletti Moja Mario, Cumbo Eduardo, Dainese Filippo, Didato Giuseppe, Fallica Elisa, Giordano Alfonso, Le Piane Emilio, Panebianco Mariangela, Piccioli Marta, Pignatta Pietro, Puligheddu Monica, Pulitano Patrizia, Ranzato Federica, Renna Rosaria, Rosati Eleonora, Vergine Stella.

**Affiliations**:

1. Department of Experimental and Clinical Medicine, Neurological Clinic, Marche Polytechnic University, Via Conca 71, 60020, Ancona, Italy
2. Epilepsy Center, Child Neurology Unit, ASST Santi Paolo Carlo, Milan, Italy
3. IRCCS Neuromed, Pozzilli, Italy
4. Department of Medical and Surgical Sciences, Magna Græcia University of Catanzaro, Catanzaro, Italy
5. Neurophysiopathology and Movement Disorders Clinic, University of Messina, Messina, Italy
6. DiBraiN, University Hospital of Bari “A. Moro”, Bari, Italy
7. Department of Biomedical, Metabolic and Neural Science, University of Modena and Reggio Emilia, Modena, Italy; Neurology and neurophysiology unit – AOU Modena, Italy
8. Department of Human Neurosciences, Policlinico Umberto I, Sapienza University of Rome, Rome, Italy

**Consensus Collaborators Group**

Audenino Daniela, Boero Giovanni, Cianci Vittoria, Coletti Moja Mario, Cumbo Eduardo, Dainese Filippo, Didato Giuseppe, Fallica Elisa, Giordano Alfonso, Le Piane Emilio, Panebianco Mariangela, Piccioli Marta, Pignatta Pietro, Puligheddu Monica, Pulitano Patrizia, Ranzato Federica, Renna Rosaria, Rosati Eleonora, Vergine Stella.

**Affiliations**

Audenino Daniela

Neurology Unit, E.O Ospedali Galliera, Genoa, Italy

Boero Giovanni

Complex Strutture of Neurology, SS Annunziata Hospital, Taranto, Italy

Cianci Vittoria

Regional Epilepsy Centre, Great Metropolitan "Bianchi-Melacrino-Morelli Hospital", Reggio Calabria, Italy

Coletti Moja Mario

Neurology Division, Ospedale degli Infermi, Ponderano (Biella), Italy

Cumbo Eduardo

Neurodegenerative Disorders Unit, ASP 2 Caltanissetta, Caltanissetta, Italy

Dainese Filippo

Department of Neuroscience, Unit of Neurology and Neurophysiology, University Hospital

of Padova, Padova, Italy

Didato Giuseppe

Epilepsy Unit, Fondazione IRCCS Istituto Neurologico “Carlo Besta”, Milan, Italy

Fallica Elisa

Department of Neuroscience and Rehabilitation, University Hospital Sant'Anna, Ferrara

Giordano Alfonso

Department of Advanced Medical and Surgical Sciences, University of Campania "Luigi Vanvitelli", Naples, Italy

Le Piane Emilio

Department of Neurology, Pugliese-Ciaccio Hospital, Catanzaro, Italy

Panebianco Mariangela

UOC Neurology and Epilepsy Centre, ARNAS Garibaldi, Catania, Italy.

Piccioli Marta

UOC Neurology, PO San Filippo Neri, ASL Roma 1, Rome, Italy

Pignatta Pietro

Neurology and Epilepsy Unit, Humanitas Gradenigo Hospital, Turin, Italy

Puligheddu Monica

Department of Medical Sciences and Public Health, University of Cagliari, Cagliari, Italy

Pulitano Patrizia

Department of Human Neurosciences, Sapienza University, Rome, Italy

Ranzato Federica

Epilepsy Center, UOC Neurology, AULSS 8 Vicenza, Vicenza, Italy

Renna Rosaria

Neurology Unit - Stroke Unit, "Cardarelli" Hospital, Naples, Italy.

Rosati Eleonora

Department Neurology 2, Careggi University Hospital, Florence, Italy

Vergine Stella

UOC Neurology, Perrino Hospital, Brindisi, Italy

Supplementary Figure 1. Flow diagram of the selection process for the literature search.


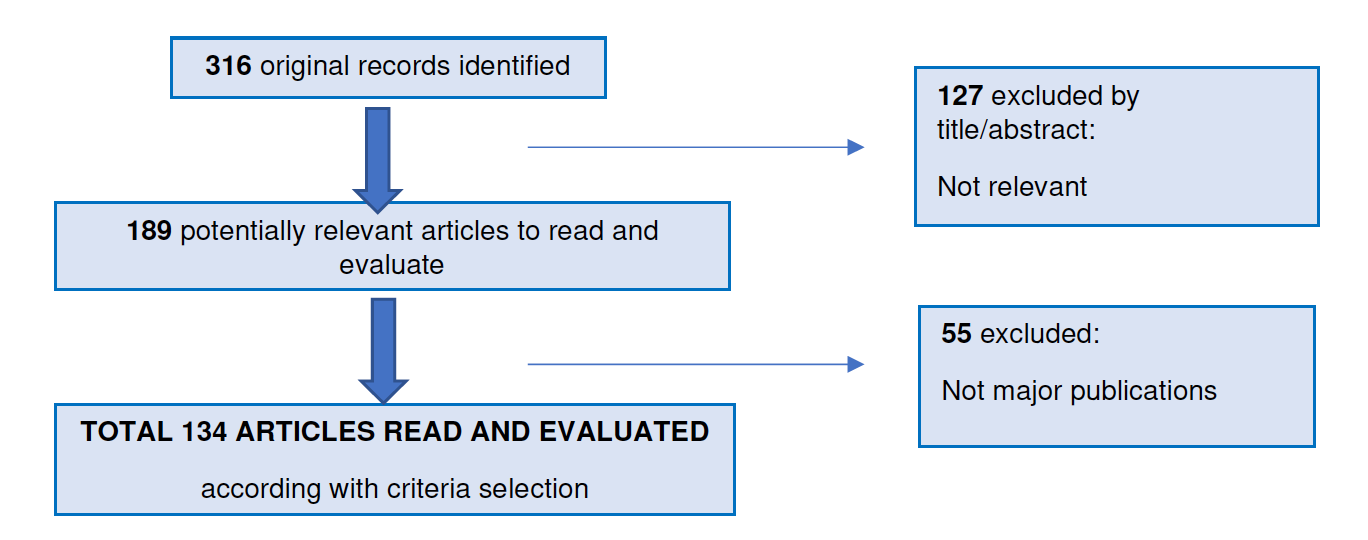

Supplement: Supplementary file 1 — Supplementary file1 (DOCX 102 KB) [file 10072_2024_7485_MOESM1_ESM.docx]
